# [Retracted] lncRNA MIAT promote cell invasion and migration in esophageal cancer

**DOI:** 10.3892/etm.2026.13084

**Published:** 2026-02-02

**Authors:** Weiguo Zhang, Qiang Chen, Caipeng Lei

Exp Ther Med 19:3267–3274, 2020; DOI: 10.3892/etm.2020.8588

Following the publication of the above paper, it was drawn to the Editor’s attention by a concerned reader that certain of the flow cytometric data shown in Fig. 3A on p. 3270 and the data panel showing the results of the ‘25 nM siMIAT’ experiment on p. 3271 were strikingly similar to data which had been already been submitted for publication to other journals that were written by different authors at different research institutes. In addition, overlapping data panels were noted with both the scratch-wound assay experiments in Fig. 6A and the western blot data in Fig. 7, suggesting that data which were intended to show the results of differently performed experiments had apparently been derived from the same original sources.

Owing to the fact that the contentious data in the above article were found to be strikingly similar to data that had already been submitted for publication elsewhere, the Editor of *Experimental and Therapeutic Molecular Medicine* has decided that this paper should be retracted from the Journal. The authors were asked for an explanation to account for these concerns, but the Editorial Office did not receive a reply. The Editor apologizes to the readership for any inconvenience caused.

